# CAR Macrophages: a promising novel immunotherapy for solid tumors and beyond

**DOI:** 10.1186/s40364-024-00637-2

**Published:** 2024-08-23

**Authors:** Jialin Lu, Yuqing Ma, Qiuxin Li, Yihuan Xu, Yiquan Xue, Sheng Xu

**Affiliations:** 1 National Key Lab of Immunity and Inflammation and Institute of Immunology, Naval Medical University/Second Military Medical University, Shanghai, 200433 China; 2 Shanghai Institute of Stem Cell Research and Clinical Translation, Shanghai, 200120 China; 3 Department of Gastroenterology, Changhai Hospital, Naval Medical University, Shanghai, 200433 China

**Keywords:** Chimeric antigen receptor, Solid tumor, Macrophages, CAR-Macrophages

## Abstract

With the advent of adoptive cellular therapy, chimeric antigen receptor (CAR)-T cell therapy has gained widespread application in cancer treatment and has demonstrated significant efficacy against certain hematologic malignancies. However, due to the limitations of CAR-T cell therapy in treating solid tumors, other immune cells are being modified with CAR to address this issue. Macrophages have emerged as a promising option, owing to their extensive immune functions, which include antigen presentation, powerful tumor phagocytosis, and particularly active trafficking to the tumor microenvironment. Leveraging their unique advantages, CAR-macrophages (CAR-M) are expected to enhance the effectiveness of solid tumor treatments as a novel form of immunotherapy, potentially overcoming major challenges associated with CAR-T/NK therapy. This review outlines the primary mechanism underlying CAR-M and recent progressions in CAR-M therapy, while also discussing their further applications.

## Introduction

Tumor immunotherapy remains a prominent and evolving field, primarily driven by the challenge of tumor immune escape. The essence of tumor immunotherapy lies in reactivating immune system’s capacity to clear foreign antigens and initiate anti-tumor immune response [1]. Furthermore, immune cells could be modified to get an increase in the ability to target tumor cells [2]. And the emergence of CAR-T cell therapy has opened up unprecedented possibilities for adoptive cellular immunotherapies. T cells armed with CARs are granted a specific role to recognize tumor associated antigens (TAAs) and exert cytocidal activity against tumor cells [3]. Nowadays, CAR-T cell therapy has shown remarkable clinical efficacy in patients with relapsed or refractory hematological malignancies [4].

While the successful application of CAR-T cell therapy heralds a promising future for people with cancer, it has also unveiled several inevitable challenges. Thus, the researchers broadened the selection of immune cells for CAR platform, extending beyond T cells to include other immune cells with safer biological functions and excellent potentials, such as NK cells.

In addition, macrophages and other cells of the myeloid lineage present themselves as viable candidates for CAR therapy owing to their potential to localize and persist within the tumor microenvironment (TME), which makes CAR-Macrophage therapy stand out [5]. As innate immune cells, macrophages possess potent phagocytic and cytotoxic capabilities, which facilitate a broad and robust immune response alongside antigen presentation, T cell activation and cytokine secretion [6]. Hence, CAR-M therapy represents a substantial advancement in the field of cellular immunotherapy.

### The strengths and weaknesses of *CAR*-T and *CAR*-NK therapies

Once T cells are grafted a defined specificity under the equipment of CARs [7], they can be engineered to target certain tumor cells that may evade T cell receptor (TCR) recognition [8]. In the last few years, substantial benefits of CAR-T cell therapy have been observed in clinical patients with chemotherapy-refractory B-cell malignancies [9]. However, the application of CAR-T therapy in solid tumors remains an challenging issue. The obstacles faced by CAR-T immunotherapy in solid tumors include insufficient CAR-T cell source, a lack of tumor-specific antigens, antigen escape, expression of heterogeneous antigen, inefficient CAR-T cell trafficking/infiltration into tumor sites and immunosuppressive TME (Table 1) [10]. Notably, the recruitment and penetration of T cells into developing dense tumor tissue appear to be particularly difficult and extensive suppressive pathways within TME would restrict T cell activation. Furthermore, a majority of patients suffered from severe therapy-associated toxicity during clinical trials. CAR-T cell activation leads to a massive release of inflammatory cytokines which may cause cytokine release syndrome (CRS) and neurotoxicity [10]. Alarmingly, CAR-T re-expansion, which can be life-threatening, was found in 3 patients [11].

**Table 1:** Comparisons between CAR-T cells, CAR-NK cells and CAR-macrophages

CAR vehicle	Superiorities	Inferiorities
T cell	•expand the range of tumor antigen targets •prevent tumor cell immune escape •promote production of proinflammatory cytokines	•GVHD •insufficient CAR-T cell source •CRS and neurotoxicity •lack of tumor-specific antigens •inefficient trafficking and infiltration into tumor sites •CAR-T re-expansion
NK cell	•no GVHD, no need for autologous NK cells •no CRS and neurotoxicity •multiple killing mechanisms besides CAR •limited lifespan, limited toxicity •rapid killing and extensive cytotoxicity of tumor cells	•require repeated administration for short half-life •inhibition of cell function by MHC molecule •poor in vivo expansion and persistence of NK cells after infusion •lack of tumor-specific antigens •inefficient trafficking and infiltration into tumor sites
Macrophage	•massive infiltration into tumor sites •phenotypic control and transformation •display enhanced phagocytic activity •less non-tumor toxicity for short time in circulation •reliable source and expansion	•high risks of insert mutations •require repeated administration •lack of tumor-specific antigens

To avoid adverse effects, the CAR strategy has been applied to other immune effector cells, such as NK cells, to evaluate its curative efficacy. The process of tumor cells elimination relies on NK cell degranulation, cytokine release, and cytotoxicity [12]. Compared with CAR-T cell therapy, no cases of CRS or neurotoxicity have been reported following CAR-NK cell therapy [13]. NK cells are accounting for 10–15% of human blood lymphocytes and lack the potential to induce graft-versus-host disease (GVHD), whereas CAR-T cell therapy necessitates the use of autologous T cells due to the risk of alloreactivity and GVHD. Furthermore, the patients requiring CAR-T cell therapy tend to have insufficient autologous T cells to support this treatment. Besides CAR-mediated targeted killing, CAR-NK cells can leverage their inherent anti-tumor properties through antibody-dependent cellular cytotoxicity (ADCC) [14], and activating receptors such as NKG2D and CD244 [15], enabling them to target cancer cells that lack target the antigens recognized by CARs.

Although CAR-NK cell therapy has an edge over CAR-T cell therapy, it has yet not come to fruition in treating solid tumors due to certain limitations (Table 1) [10]. NK cells often fail to achieve adequate in vivo expansion and persistence after infusion [16], which may necessitate repeated infusions. With the administration of various cytokines, such as IL-15 [17], IL-18 [18], IL-21 [19], the in vivo persistence of NK cells will be improved, but the undesirable effects including autonomous proliferation of NK cells and their transform into leukemia also occurred under the stimulation of these cytokines [20].Furthermore, MHC molecules may bind to inhibitory killer Ig-like receptors (KIRs) present on the surface of CAR-NK cells, resulting in functional inhibition of NK cells [21]. On account of the impossibility of the rearrangement of receptor genes and inevitable exhaustion of NK cells when exposed to the immunosuppressive tumor microenvironment, adoptively transferred NK cells cannot develop immunological memory, which results in a failure of long-term protection [16]. In addition, some limitations observed in CAR-T cells are also present in CAR-NK cells, such as the rarity of tumor target antigen, challenges in infiltrating into tumor sites, post-infusion complications and so on.

### Macrophage: a promising immunotherapy candidate

Macrophages are a type of innate immune cells that exert a wide range of functions to pathogen clearance and the maintenance of tissue homeostasis [22]. Their Diversity and plasticity are significant characteristics that enable the development of various therapeutic strategies. Macrophages exhibit dual functions within TME through different subtypes [8]. Tumor-associated macrophages (TAMs), a complex and heterogeneous population of cells, undergo dynamic changes in phenotypes and functions in response to TME stimuli [23]. These include “classically activated” (M1)macrophages, which feature a pro-inflammatory phenotype and “alternatively activated” (M2) macrophages, which adopt an anti-inflammatory phenotype [24]. M1 macrophages are equipped with anti-tumorigenic properties that can efficiently eliminate tumor cells through phagocytosis and cytotoxicity, while M2 macrophages on the other hand help to promote tumor angiogenesis, immunosuppression, growth, and metastasis [25].

**Fig. 1:**
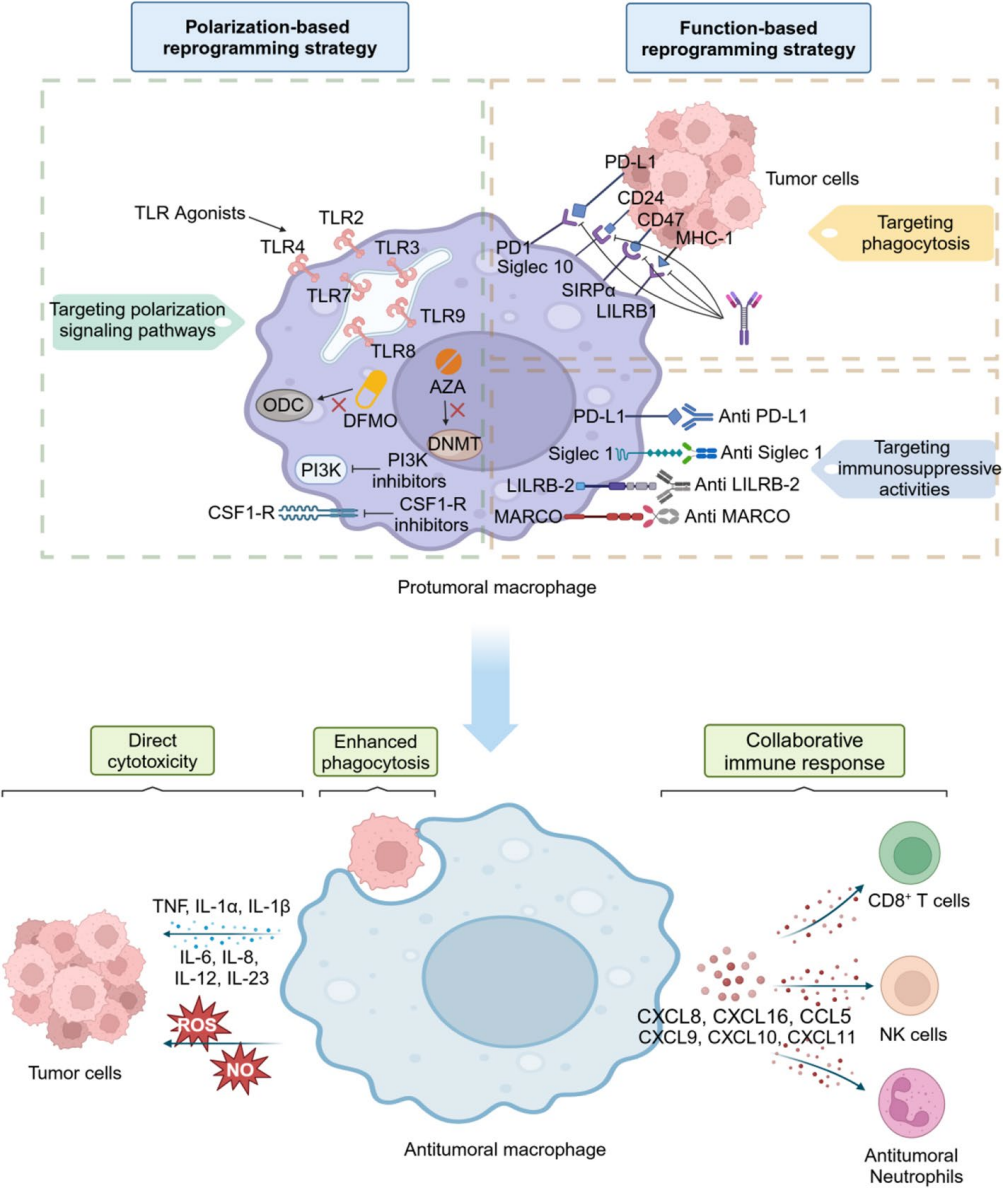
Therapeutic strategies aiming at altering the phenotype of TAMs from a protumoral to an antitumoral state. TAM reprogramming strategies fall into two main categories: polarization-based reprogramming and function-based reprogramming of TAMs. Polarization-based reprogramming strategy primarily targets macrophage polarization signaling pathways, using TLR agonists, CSF1-R inhibitors, PI3K inhibitors and certain epigenetic inhibitors. Function-based reprogramming of TAMs aims at specific TAM functions, such as phagocytosis by targeting “do not eat me” signals SIRP1α-CD47, LILRB1-β2M, Siglec10-CD24, and PD1-PDL1. Function-based reprogramming of TAMs can also target macrophage immunosuppressive activities by inhibiting Siglec1, PD-L1, MARCO and LILRB2. Macrophage reprogramming results in the secretion of proinflammatory cytokines and ROS, improvement of phagocytic ability and macrophage-mediated immune promotion through CD8 + T cells, NK cells and neutrophils

Considerable evidence has indicated that during T helper 1 (Th1) based immunologic responses, macrophages undergo M1­type activation, secreting cytokines such as tumor necrosis factor (TNF), IL-1, IL-6,IL-8, and IL-12 [26]. These activated macrophages play crucial roles in phagocytosing and destroying tumor cells (Fig. 1). M1 macrophages produce tumor killing molecules, including reactive oxygen species (ROS) and toxic nitric oxide (NO), which exert direct cytotoxic effects on tumor cells [27, 28]. In addition, M1 macrophages promote the infiltration and activation of effector T cells by producing chemokine CXCL10, TNF, and other cytokines that enhance immune responses [29]. Through these mechanisms, various tumor cells can be damaged both in vitro and in vivo. Conversely, during Th2 response, macrophages undergo M2 type activation, characterized by the secretion of IL-4,IL-10, IL-13 and TGF-β, which play vital roles in promoting the growth of solid tumors and suppressing immunologic attack [30]. The high expression of Arginase 1 (Arg1) in M2 macrophages has been shown to mediate polyamine synthesis and collagen formation, as well as to stimulate tumor cell growth and repair damaged tissue [31]. Additionally, M2 macrophages have the capacity to produce the cytokines that enhance permeability of tumor vessels, thereby accelerating tumor metastasis [32]. In contrast to the abundant expression of MHC II and co-stimulatory molecules, such as CD80 and CD86 in M1 macrophages [33], the expression of MHC II in M2 macrophages, although considerable, is insufficient to make efficient antigen presentation [34].

Although M1 macrophages have demonstrated anti-tumor abilities, the majority of TAMs exhibit behavior characteristic of M2 macrophages, which promote tumor progression [35]. Thus, reprogramming of TAMs may be an effective and promising strategy for macrophage-based immunotherapy.

Several solutions have been explored to make TAMs play a cytotoxicity role within TME. TAM reprogramming strategies fall into two main categories: polarization-based and function-based reprogramming of TAMs [36] (Fig. 1).

Polarization-based reprogramming strategy primarily inhibits the signaling pathways involved in macrophage polarization. TLR agonists have been identified as potent immunostimulatory molecules capable of inducing M1 macrophage repolarization [37], while the CSF1/CSF1-R signaling pathway between tumor cells and macrophages is responsible for the phenotypic transition to immunosuppressive M2 phenotype [38]. Blocking this pathway with the CSF1-R inhibitor PLX3397, or GW2580 [39, 40] significantly reduced both the proportion of M2 macrophages and the total number of TAMs [41], thereby allowing M1 macrophages to become the predominant component of TAMs and exert anti-tumor efficacy [42]. Within the TME, M2 macrophages promoted tumor invasiveness via the PI3K-PKB/Akt signaling pathway [43]. And treatment with the PI3K inhibitor LY294002 resulted in an increase in the expression of M1-type proteins [44]. The application of molecular targeted drugs could also affect the phenotype of macrophages. Epigenetic DNA methyltransferase (DNMT) inhibitor 5-Azacytidine (AZA) and an ornithine decarboxylase (ODC) inhibitor α-difluoromethylornithine (DFMO) in combination dramatically reduced the number of M2-polarized macrophages and increased tumor-killing M1 macrophages, which significantly reduced tumor burden and prolonged survival [45].

Function-based reprogramming strategies involve promoting the phagocytic function of TAMs and inhibiting their immunosuppressive activities. Regarding the regulation of phagocytosis (Fig. 1), several signaling pathways can be targeted, including the programmed cell death protein (PD)-1/PD-L1signaling pathway [46], CD47-SIRPα mediated ‘don’t-eat-me’ signal [47], the leukocyte immunoglobulin-like receptor subfamily B member 1 (LILRB1)-β2-microglobulin (β2M) signaling pathway [38], and the sialic-acid-binding Ig-like lectin 10 (Siglec10)-CD24 signaling [48]. These pathways inhibit the phagocytosis of macrophages and mediate the occurrence of tumor immune escape. Once these signaling pathways were blocked, macrophages would be reactivated, strengthening their phagocytic function. To inhibit the immunosuppressive activities of TAMs (Fig. 1), TAM-preferentially expressed inhibitory receptors such as macrophage receptor with collagenous structure (MARCO) and leukocyte immunoglobulin-like receptor-B2 (LILRB2) [49] can be good targets. The introduction of MARCO antibodies [50] and JTX-8064, which disrupted the binding of LILRB2 to HLA-G on tumors [51], has been shown to promote anti-tumor immunity. Similar effects can also be achieved by targeting Siglec-1 and PD-L1 on macrophages [52, 53].

### *CAR*-M therapy emerges on demand

The extracellular matrix (ECM), formed by the highly organized interactions of a variety of macromolecular proteins, fiber molecules, proteoglycans, glycoproteins and glycosaminoglycans, acts as a physical barrier to most anti-tumor therapies, including CAR-T and CAR-NK therapies [54]. In contrast, macrophages are able to infiltrate the TME by secreting a wide range of matrix metalloproteinases (MMPs) that are engaged in extracellular matrix degradation [55]. Macrophages are a key source of MMPs, capable of degrading nearly all components of the ECM and basement membrane [56]. Numerous studies have demonstrated that macrophages exist massively in multiple tumors [57], including renal cancer, melanoma, and colonic-carcinoma. Under the combined influence of hypoxia and cytokines, such as CCL2, CCL5 and CSF1–-produced by tumor cells, fibroblasts, endothelial cells, or TAMs themselves, macrophages will be largely recruited to hypoxic tumor compartments. Once in the hypoxic environment, the expression of the receptor of CCR2, CCR5, and neuropilin-1 (NRP1) on the macrophage is down-regulated, effectively trapping them in the hypoxic TME [58]. Klichinsky et al. evaluated the trafficking, biodistribution and persistence of CAR-M in five different solid tumors, confirming its substantial existence at the tumor site [59]. Based on the fact that macrophages have the ability to infiltrate tumor tissue in large numbers, CAR-M therapy holds significant promise in immunotherapy (Table 1).

Another challenge to the widespread adoption of CAR-T therapy is the great tendency to induce CRS and neurotoxicity. In contrast, severe CRS rarely occurs in CAR-M therapy, attributable to its limited expansion potential and transient persistence in peripheral blood [60].

In addition to the aforementioned benefits, CAR-M improves antigen presentation and promotes a greater cytotoxic response from T cells, demonstrating a superior ability to kill tumors. The merits of CAR-M therapy pave the way for extensive research into CAR therapies, leading to the development of various CAR generations tailored to meet specific clinical needs.

### The mechanisms of *CAR*-M immunotherapy

**Fig. 2:**
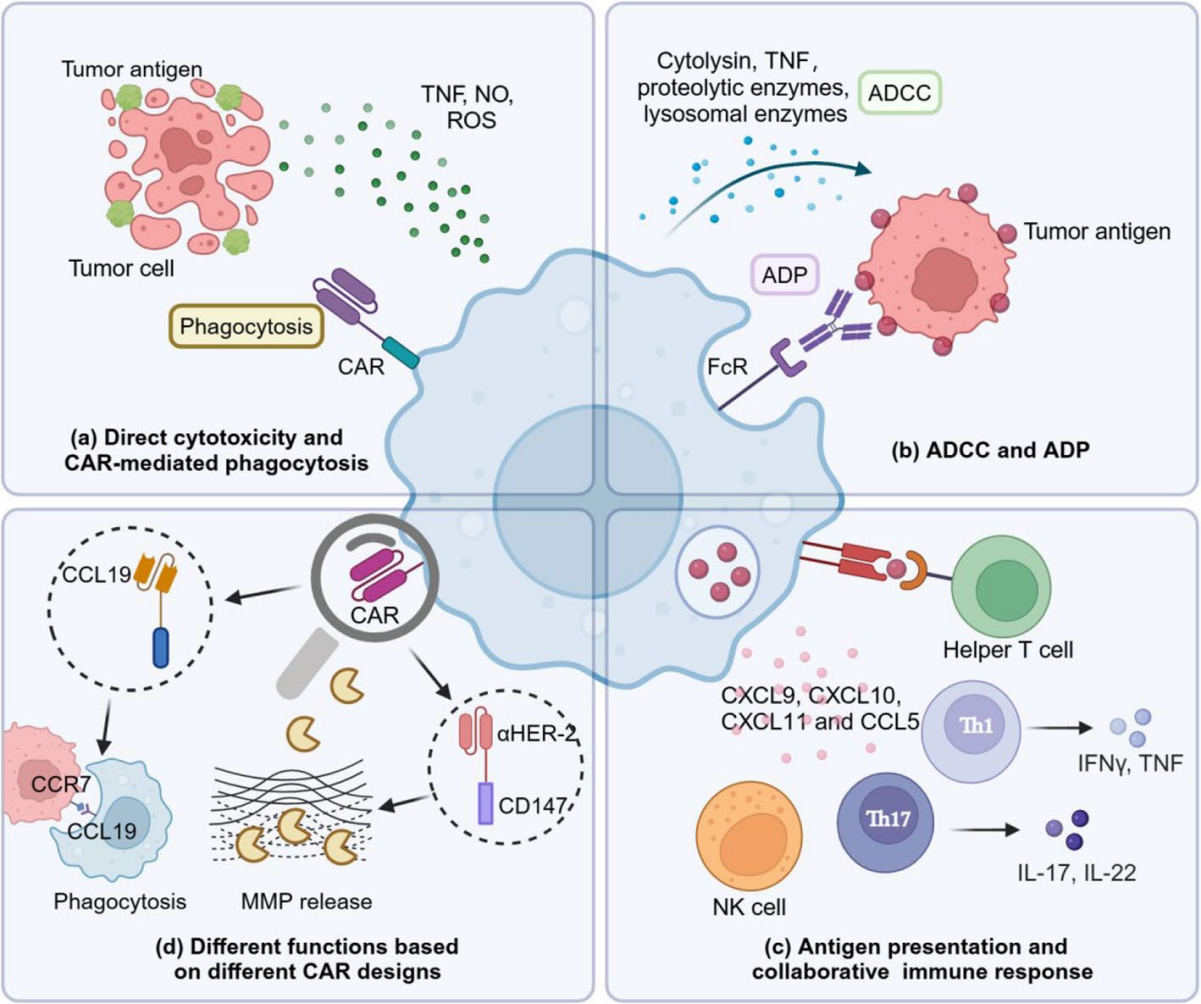
Mechanisms of CAR-M. The killing of tumor cells by CAR-M can be reflected in many aspects. **a** CAR-M mediate direct cytotoxic effects by releasing TNF, NO and ROS. **b** CAR-M exert ADCC and ADP effects through the binding of FcR on its surface to Ab coated on the tumor cells. **c** Collaborative immune response can be achieved when CAR-M presents tumor antigens to helper T cells and recruit other immune cells. **d** CAR-M can play different roles depending on the CAR design. CCL19-CAR-M—promotes the engulfment of CCR7-positive tumor cells, which can slow tumor progression and metastasis. CD147-CAR-M—triggers MMPs production to degrade ECM, and promotes more immune cell infiltration

Substantial experiments have displayed that CAR-M therapies possess the capacity to exterminate tumor cells in vitro and in preclinical in vivo models. And the identification and killing mechanism are multiplefaceted. CAR-M can mediate cytotoxicity to tumor cells directly. LPS-activated macrophages will secrete harmful substances that disintegrate tumor cells, including TNF, NO and ROS (Fig. 2a). These are the primary tumorcidal effector molecules of CAR-M, playing complementary and synergistic roles in anti-tumor activities. TNF can directly induce apoptosis in tumor cells. Duan et al. designed vascular endothelial growth factor receptor-2 (VEGFR2)-targeting CAR-M, which was activated by TLR4 and/or IFN-γ receptors. The study has showed that CAR-M activated by VEGFR2 generated substantial amounts of TNF-α, inhibiting tumor growth effectively [61]. NO can diffuse to nearby tumor cells and react with oxygen radicals, resulting in peroxynitrite generation, nitrosylation of proteins, and eventual apoptosis of the tumor cells [62]. Moreover, NO may lead to the inhibition of many metabolic activities of tumor cells, such as mitochondrial respiration, DNA replication and so on, giving rise to significant iron loss in tumor cells and ultimately causing cell death. It has been shown that NO can promote TNF to kill tumor cells that are insensitive to TNF. At the same time, TNF can also induce Caspase-dependent apoptosis by accelerating ROS release [63]. In addition, ROS released by CAR-M can induce DNA damage and genetic instability, which lead to tumor apoptosis and necrosis [64]. A recent study demonstrated that ACOD1-knockout CAR-iMac can be consistently maintained in a pro-inflammatory state, resulting in increased production of ROS and enhanced phagocytic activity [65].

Furthermore, CAR-M can mediate direct cytolysis of tumor cells through antibody dependent cellular cytotoxicity (ADCC) and antibody dependent phagocytosis (ADP) (Fig. 2b). Through the Fc receptor (FcR) expressed on its surface, CAR-M recognizes and binds to the antibodies coated on the tumor cells. It can then either destroy the tumor cells by releasing cytotoxic substances such as cytolysin, proteolytic enzymes, lysosomal enzymes or TNF (ADCC), or it can directly phagocytose tumor cells (ADP).

The wide communication between macrophages and other immune components also plays a critical role in tumor clearance. Some researchers have observed that CAR-M not only functioned as antigen-presenting cells that present tumor antigens to prime T cells, but also increased intratumoral infiltration of tumor infiltrating cells, such as antitumoral neutrophils, Th1, Th17, cytotoxic T cells (CTL) and NK cells by secreting CXCL8, CXCL9, CXCL10, CXCL11 and CCL5 [1] (Fig. 2c). What’s more, single-cell sequencing studies have revealed that CAR-M can maintain the M1 phenotype in tumor tissue, with high expression of inflammation-related genes [59]. Activated M1 macrophages release pro-inflammatory cytokines, such as IL-1β, IL-6, IL-12, IL-18 and TNF, which create a pro-inflammatory microenvironment and activate T cell responses, indicating a systemic and collaborative immune function [59, 66, 67]. CAR-Ms targeting HER-2 and CD47, respectively, have been reported to act synergistically with CD8^+^ T cells, prompting them to secrete various cytokines that attack ovarian cancer cells in vitro [68].

Depending on the CAR design, macrophages can also function in other ways (Fig. 2d). Tumor cells expressing CCR7 could interact with CCL21 produced by lymphatic endothelial cells, resulting in lymphatic metastasis of tumor cells [69]. To mitigate tumor invasion and progression, CAR has been engineered to direct macrophages toward CCR7-positive tumor cells, which are part of a lipid droplet high (LD^hi^)CCR7^hi^ immunosuppressive cell population [66] (Table 2). The researchers selected CCL19, a natural ligand of CCR7, to constitute CCR7-specific CARs instead of antigen-recognition domain scFvs [66]. The CCR7-targeting CAR-M presented antigen-specific cytotoxicity to combat tumor cells, reducing the tumor metastasis and prolonging overall survival in breast cancer [66]. In addition, HER-2 CAR-macrophages modified with internal signaling domain of CD147 are aimed at the tumor ECM rather than tumor cells themselves [56]. CD147 is a membrane molecule involved in cell–matrix interactions, and its activation can stimulate the expression of MMPs to degrade collagens [70]. The destruction of ECM disrupts the physical barrier of tumor, facilitating the infiltration of immune cells. CAR-HER-2-CD147 specifically activates MMP expression in CAR-M within the TME upom recognition of the tumor antigen HER-2, with no improvements in killing, phagocytosis, ROS production and cytokine release [56]. However, following the infusion of CAR-147 macrophages, tumor collagen deposition decreased and T cells infiltrated into the tumor more actively [56].

**Table 2:** Different structures of CAR with different traits and purposes

Object	Macrophage source		Target antigen	Extracellular domains	Intracellular domains	CAR delivery method	Unique advantages	Common features	Ref
Targeting tumor cells	Murine	J774A.1 murine macrophages	CD19, CD22	scFv	CD3ζ	the lentiviral vector		·have ITAMs, which initiate phagocytosis	[71]
					FcRγ(+ PI3K)		·promote the phagocytosis of the entire cancer cell		
					Megf10		·can target different antigens ·improve the ability to ingest whole cancer cells		
		Raw264.7 monocyte/macrophages	CCR7	CCL19	TLR-2, TLR-4, TLR-6	the lentiviral vector		·have phagocytic ability	[66]
					MerTK		·have the most efficient cytotoxicity ·promote the phagocytosis of CCR7-positive tumor cells		
					4-1BB-CD3ζ				
			GPC3	scFv	CD3ζ	LNPs	·The ITIMs-lacking Siglec-G expressed on the surface of macrophages competitively binds to CD24, blocking the binding of CD24 to natural Siglec-G, thereby reversing the immunosuppression of macrophages and significantly enhancing the phagocytosis of CAR-Ms		[72]
			ErbB2	scFv	CD3ζ	DNA nanocomplex	·DNA nanocarriers can perform in situ genetic editing on ErbB2-specific CAR-Ms within tumors to guide their phagocytosis of tumor cells ·RP-182 in nanoparticles can facilitate the conversion of M2-like TAMs into anti-tumor M1-like phenotypes		[73]
		RAW 264.7 macrophages and bone marrow-derived macrophages	SasA	scFv	CD3ζ	peptide nanoparticles containing plasmid DNA	·have elevated phagocytotic activity against MRSA ·have potent bactericidal activity in vivo and in vitro		[74]
		bone marrow-derived macrophages	uPAR	scFv	CD3ζ	the adenoviral vector pDC315-CAR and pBHGlox E1,3Cre	·have high phagocytic activity against HSCs		[75]
	Human	Induced pluripotent stem cells (iPSCs)	CD19	scFv	CD86 + FcRγ	the lentiviral vector	·possess M2 phenotype and can polarize toward M1 when treated with IFN-γ	·iPSCs are able to generate adequate CAR-Macrophages ·CAR-iMac cells can expand ·perform antigen-specific phagocytosis	[67]
					CD3ζ + TIR		·enhance cytotoxic activity to tumor cells ·maintain M1 polarization state ·inhibit M2 phenotype ·improve antigen presenting ability		[76]
					FcRγ + CD19		·demonstrate enhanced phagocytosis and cytokine secretion on co-culture with CD19^+^ Raji cells ·up-regulate pro-inflammatory-related genes		[77]
			Glioblastoma	chlorotoxin	CD3ζ		·show higher secretion of IL-6 and TNF-α ·display an enhanced anti-cancer cell activity		[78]
			PSCA	scFv	CD3ζ		·has a suicide switch that is capable of removing unwanted CAR-iMacs in vivo in the event of uncontrolled or unsafe CARimacs expansion		[79]
		Human THP-1 cell line	HER-2	scFv	CD3ζ	an Ad5f35 vector	·can maintain M1 phenotype in vivo and in vitro	·direct anti-tumor phagocytic activity	[59]
			CD47	scFv	CD3ζ	the lentiviral vector	·collaborate with β-CD LNPs to enhance phagocytosis		[80]
			human mesothelin	scFv	4-1BB-CD3ζ	mRNA transfection	·reduce target-specific off-tumor toxicity		[81]
Targeting ECM	Murine	Raw264.7 monocyte/macrophages	HER-2	scFv	CD147	the lentiviral vector pLenti6/V5-D-TOPO	·do not exhibit engulfment ·upregulate the expression of MMPs ·degrade ECM and prompt more immune cells infiltration		[56]

Above all, the anti-tumor effect of CAR-M is not merely a singular effort; rather, it represents a comprehensive immune response aimed at eliminating tumor cells through multiple mechanisms, engaging the entire immune system in the fight.

### Cell Sources of *CAR*-M

In addition to phenotypic control and transformation, a reliable source and expansion of macrophages are essential for the clinical applications of CAR-M therapies. Although macrophages can be collected from model cell lines such as THP1 and Raw 264.7, or from immortalized murine bone marrow-derived macrophages, there remains a critical need for a sufficient and scalable source of primary human macrophages is still a necessity for clinical translation. This necessity arises because model cell lines are unsuitable for clinical settings, and efficiently engineered macrophages cannot be guaranteed. Clinically applicable macrophages are typically derived from peripheral monocytes and induced pluripotent stem cells (iPSCs).

As to ex vivo manufacturing of monocytes, MaxCyte’s MCY-M11 electroporationally transfects unamplified autologous peripheral blood mononuclear cell (PBMC) with CAR-mRNA encoding human anti-mesoderin and differentiates them into CAR-M subsequently [82]. This CAR therapy allows for instantaneous expression without the need for viral vectors or cell expansion, facilitating rapid manufacturing and timely delivery back into the patient. Approximately 550 million blood monocytes can be obtained from a single donor via leukapheresis [83]. And with the addition of recombinant human GM-CSF, the monocytes continue to proliferate [84], providing a substantial source of macrophages. Different from the method of transfection followed by differentiation mentioned above, Klichinsky et al. gained primary human macrophages from peripheral blood CD14^+^ monocytes first [59]. Under the influence of GM-CSF, CD14^+^ monocytes were promoted to differentiate into macrophages and favored the pro-inflammatory M1 phenotype [85]. Subsequently, Ad5f35 was transduced into these macrophages for the expression of anti-HER2 CAR efficiently.

CAR-M can also meet a large number of cell requirements with iPSCs. The researchers derived iPSCs from PBMC of a healthy donor and isolated single iPSC clones [67]. IPSCs are then utilized for the expression of CAR with lentivirus, and subsequently induced to differentiate into macrophages. The iPSC-derived CAR-macrophages (CAR-iMac) can be available for multiple times in a month, with the final yield 50 times more of the initial iPSCs, and thus providing a sufficient source of macrophages ready for clinical use [67]. The current modified protocol already allows for a series of 18–25 harvests with single harvest yields of up to 6 × 10^8^cells [86]. There is no upper limit on the number of macrophages that can be produced if the process is appropriately scaled. More surprisingly, the researchers have developed a monolayer-based system for the efficient production of about 6,000 macrophages from a single human pluripotent stem cell (hPSC) within 3 weeks [87]. Furthermore, macrophage exhibits a lower incidence of GVHD compared to T cells, making them suitable for collection as off-the-shelf universal cells for unforeseen needs. CAR-iMac possess the potential to engage in antigen-dependent phagocytosis. In the absence of specific antigens, CAR-iMac remain inactive and maintain an anti-inflammatory M2 phenotype [88]. However, when tumor-associated antigen targeted by CAR are present, M1 pro-inflammatory cytokines are released, exerting anti-tumor effects [67]. Nowadays, CAR-iMac have been applied in the treatment of CD19-positive leukemia and glioblastoma, demonstrating significant therapeutic potential [77, 78]. And new research has showed that human iPSC-derived CAR macrophages targeting prostate stem cell antigen (PSCA) exerted enhanced and specific phagocytosis of PSCA^high^ pancreatic tumor cells [79].

However, several challenges still need to be addressed in preclinical settings. As previously mentioned, the phenotype and anti-tumor potential of CAR-iMac must be comparable to that of natural macrophages [89]. Thus, the polarization of iPSC-derived macrophages warrants attention to advance CAR-M therapy. Furthermore, safety testing of iPSC-derived cells remains crucial. Given that iPSC cells have the potential to proliferate indefinitely, undifferentiated iPSC cells pose a significant risk of malignant transformation when imported into patients. Consequently, safety screening or genetic engineering strategies need to be established to minimize this risk. For example, suicide genes, such as the inducible caspase-9/chemical inducer of dimerization (iC9/CID) system, can be employed to initiate a caspase cascade that eliminates iPSCs through activation of iC9 by CID [90].

### Generations of *CAR*-Macrophages

Traditional CARs consist of three functional domains: an extracellular domain, a transmembrane domain and an intracellular domain [3]. The extracellular domain is composed of scFv, which is a single chain variable fragment of a monoclonal antibody responsible for recognizing and binding TAAs, and a hinge that serves as a connection. And the intracellular domain comprises both the signal transduction domain and costimulatory domain [91].

Current efforts to engineer CAR-M have demonstrated that CAR design principles applicable to T cells are also relevant for macrophages. The CAR design has been iteratively refined to meet different purposes. Thus, the construction of CAR-M deserves ongoing optimization design and functional evaluation to customize diverse therapeutic needs.

**Fig. 3:**
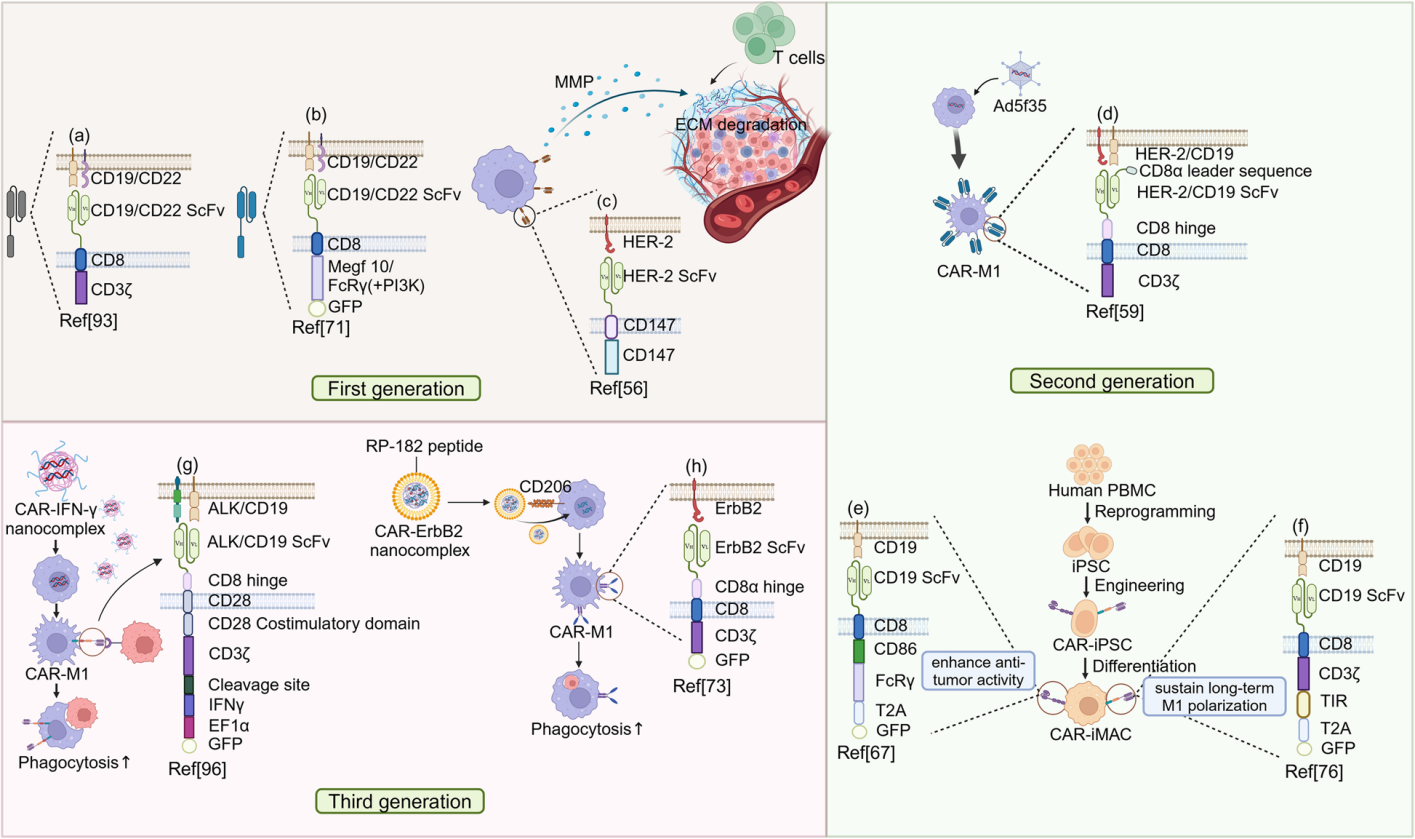
The various generations of CAR-M. According to CAR structural design, first-generation CAR-M includes **a**, **b** and **c**. **a** The inclusion of CD3ζ as an intracellular domain can promote the capacity of CAR-M. **b** The intracellular domain Megf10 significantly enhanced phagocytosis. And the whole cell eating ability was further promoted by the addition of PI3K. **c** The intracellular CD147 signal led to the degradation of ECM, thereby recruiting more T cells to the TME. Second-generation CAR-M includes **d**, **e** and **f**. **d** Ad5f35 was utilized to transfer the anti-HER2 CAR into macrophages, which tilted macrophages toward the M1 phenotype. (**e**) IPSC-differentiated CAR-expressing macrophages provided a sufficient source of CAR-M. The anti-tumor activity of macrophages expressing CD86-FcRγ-CAR was significantly enhanced, **f** while macrophages with CD3ζ-TIR-CAR can sustain long-term M1 polarization. Third-generation CAR-M includes **g** and **h**. **g** IFN-γ was added to the intracellular domain via CAR-IFN-γ nanocomplex, markedly enhancing anti-tumor efficacy. **h** Nanocomplex carrying RP-182 peptide not only promoted the macrophage phenotype to M1 but also delivered the CAR-ErbB2 gene to macrophages, thereby improving their phagocytosis

Similar to the structural design of CAR in CAR-T cells, first-generation CAR-M are designed to improve its ability to target tumor-specific antigens and perform phagocytosis [92]. In CAR-T cells, CD3ζ, whose immunoreceptor tyrosine-based activation motif (ITAM) is phosphorylated to associate with SH2 domains of ZAP-70 in T cells, is consistently selected as the intracellular domain of CAR. Although CAR-M do not express ZAP-70, the ITAM of CD3ζ can bind to SH2 domains of Syk kinase [93]. While the inclusion of CD3ζ as an intracellular domain can promote the capacity of CAR-M, its non-macrophage origin may not fully leverage the potential of macrophages (Fig. 3a). Thereafter, the ITAM-containing Fc receptor common γ-chain (FcRγ) and multiple EGF-like-domains protein 10 (Megf10) are chosen as the intracellular domain, which has increased the phagocytosis of CAR-M [71]. What’s more, to promote the internalization of large targets, PI3K signaling motifs are also introduced, which increases the frequency of the engulfment of the whole cell by threefold [94] (Fig. 3b and Table 2). Distinctively, unlike the direct intention to trigger phagocytosis, HER2-CD147 CAR-macrophages indirectly inhibit the tumor growth by recruiting additional T cells [56]. After recognition of HER2 on tumor cells, intracellular CD147 signal activates MMP production to degrade the dense collagen-based tumor matrix, which promotes infiltration of T cells into tumor cells, thereby achieving therapeutic effect [56, 95] (Fig. 3c).

Second-generation CAR-M aims to improve antigen presentation and T cell activation ability after infusion [92]. To achieve these objectives, it is desirable to induce and maintain the pro-inflammatory M1 phenotype of CAR-M while massively expanding them to achieve optimal quantitis for infusion. To address the plasticity of CAR-M, Klichinsky et al. utilized an adenoviral vector (Ad5f35) to transfer anti-HER2 CAR into human macrophages (Fig. 3d and Table 2). Regardless of CAR expression, adenovirally transduced macrophages inclined to undergo classical activation pathways to become the M1 phenotype and could be sustained for at least 40 days [59]. As for the amplification of CAR-M, Zhang et al. established CAR-iPSC-differentiated CAR-expressing macrophages to generate sufficient CAR-iMac, which can expand over 50-fold and persist for around 30 days [67] (Fig. 3e and Table 2). Recently, CD3ζ-TIR-CAR has been created, which integrates intracellular CD3ζ and TIR domains in tandem [76]. After the recognition of LPS by TLR4, which is widely expressed on the surface of macrophages, the intracellular TIR domain triggers downstream signaling, promoting the polarization of macrophages to a M1-like proinflammatory state via the NF-κB pathway, while inhibiting the M2 phenotype. Then CD3ζ-TIR-CAR was transduced into iPSCs to obtain the second generation of CAR-iMac [76] (Fig. 3f and Table 2).

Third-generation CAR-Ms have evolved significantly to markedly enhance anti-tumor efficacy, particularly through the integration of nanobiotechnology and in vivo reprogramming [92]. Kang et al. managed to utilize macrophage-targeting nanocarriers to transfer plasmid DNA encoding CAR-interferon-γ into macrophages in vivo (Fig. 3g). The expression of IFN-γ gene facilitates the reprogramming of CAR-M polarization from M2 to M1, which raises the level of CAR-mediated tumor phagocytosis and anti-tumor immunomodulation [96]. However, the random distribution property of nanocarriers impairs the efficient and complete uptake by macrophages at the tumor site. Recent advancements have introduced a synthetic universal DNA nanocarrier featuring RP-182 peptides located in the nanoshell, designed specifically to target macrophages [73] (Fig. 3h). RP-182 could bind to and activate CD206, subsequently reprogramming TAMs to adopt a M1-like antitumor phenotype, thus achieving better locoregional delivery [97]. Utilizing RP-182, ErbB2 CAR gene-laden DNA nanocomplex has successfully pinpointed ErbB2-specifc CAR macrophages at the tumor site and directed their phagocytic activity towards tumors [73]. And fortunately, the clinical application of this synthetic DNA nanocarrier for brainstem gliomas has gained some therapeutic effects, propelling the development of CAR-M therapy.

### Struggle for the delivery of CARs into macrophages

Once the specific CAR gene is transferred into macrophages, the phagocytic activity against tumor cells and the capacity for antigen presentation are proved to be improved [98]. However, delivering CARs and other genes to macrophages remains a challenging task. Myeloid cells are sensitive to non-self nucleic acids on account of the existence of nucleic acid-sensing receptors [99], which render macrophages and monocytes resistant to genetic manipulation [1]. With advancements in genetic technology, several viral and non-viral strategies have been implemented.

Myeloid cells such as monocyte-derived macrophages are relatively refractory to the infection of HIV-1-derived lentivirus owing to the myeloid-specific restriction factor, SAMHD1, which prevents efficient reverse transcription by hydrolyzing cellular dNTPs [100]. However, HIV-2 and viruses from the simian immunodeficiency virus (SIV) lineage, which encode Vpx, a viral accessory protein, can transduce myeloid cells more readily. Vpx counteracts SAMHD1 by mediating its degradation [101]. Considering the absence of Vpx in HIV-1, the use of Vpx-containing lentiviral particles provides greater opportunities for achieving efficient transduction of myeloid cells.

Apart from the application of lentiviral vectors, adenoviral vectors can effectively achieve transient yet high-level gene delivery and expression [102]. Although the majority of blood or bone marrow-derived cells lack the coxsackie and adenovirus receptor, which serves as the primary docking site for the conventional Ad5-based vectors, CD46 is highly expressed in monocytes and macrophages, acting as a cellular attachment receptor for group B adenoviruses, such as Ad35 [103]. Ad5f35 vectors, created by replacing the fiber gene of Ad5 with that of Ad35, engage a high efficiency of HER-2 expression in human macrophages [59]. Furthermore, CAR expression could be maintained for at least 1 month in vitro and 62 days in vivo. Remarkably, Ad5f35 even has the capacity to impart a sustained proinflammatory (M1) phenotype, which triggers the anti-tumor immune response [1, 59].

Thus, in regard to viral strategies, both lentiviral vectors and adenoviral vectors serve as effective platforms for delivering genetic material and facilitating the efficient transfer of CAR. While AAVs are commonly utilized in CAR-T and CAR-NK cells, their application in CAR-macrophages is relatively rare [104].

Non-viral strategies are also useful for delivery of genes into monocytes and macrophages. The recent development of mRNA vaccines suggests that the mRNA modification seems to be a plausible way to deliver CARs in CAR-macrophages [105]. To optimize the transient delivery of mRNA to the primary human monocytes and macrophages, which are typically resistant to genetic manipulation, various factors such as mRNA doses, nucleotide modifications, and different carriers have been taken into consideration [105]. Notably, the use of mRNA electroporatively transduced CAR-M decreased target-specific off-tumor toxicity, attributed to the transient nature of CAR expression [81].Furthermore, CAR-Ms transfected by mRNA have been reported to exhibit target-specific killing of human mesothelin expressing mouse ovarian tumor cells in vitro [81].

So far, nanoparticles have also been a good vehicle for non-viral gene delivery. Lipid nanoparticles (LNPs) facilitated the delivery of mRNA by addressing the inherent instability of naked mRNA, which is prone to rapid degradation by nucleases, thereby achieving high-efficiency delivery. The use of LNPs in gene delivery has achieved promising success in the treatment of hepatocellular carcinoma (HCC) [72]. Consequently, mice treated with such LNPs generated glypican-3 (GPC3) CAR-Ms significantly in vivo, reduced tumor burden and increased survival time in an HCC mouse model. Following systemic administration, LNPs formulated with ionizable lipid PPZ-A10 were developed to preferentially target hepatic macrophages for mRNA delivery [106]. Furthermore, nanoparticles containing DNA also were utilized to deliver CAR gene into macrophages, thereby constructing CAR-Ms to engulf specific cells or bacteria [73, 74].

Currently, CAR-M gene transfer is still primarily carried out through virus transfection, which poses a high risk of insert mutations with potentially adverse consequences. To solve this thorny issue fundamentally, the researchers have made extensive efforts to devise innovative solutions. A CRISPR-Cas9 genome targeting system has been developed that does not rely on viral vectors, allowing rapid and precise site-specific insertion of DNA molecular templates [107]. This strategy is now applied to CAR-T gene editing, accurately modifying genetic alteration in T cells and replacing the endogenous TCR with a new TCR that is better positioned to target tumor-surface antigen. The successful application of CRISPR-Cas9 genome targeting system in CAR-T implies the potential for its implementation in CAR-M, thereby avoiding the undesirable effects caused by mutated genes [108].

### Novel approaches to polish *CAR*-M therapy

CAR-M breaks through the limitations of CAR-T therapy, and novel approaches utilizing CAR-M hold significant potential for making further progress.

**Fig. 4:**
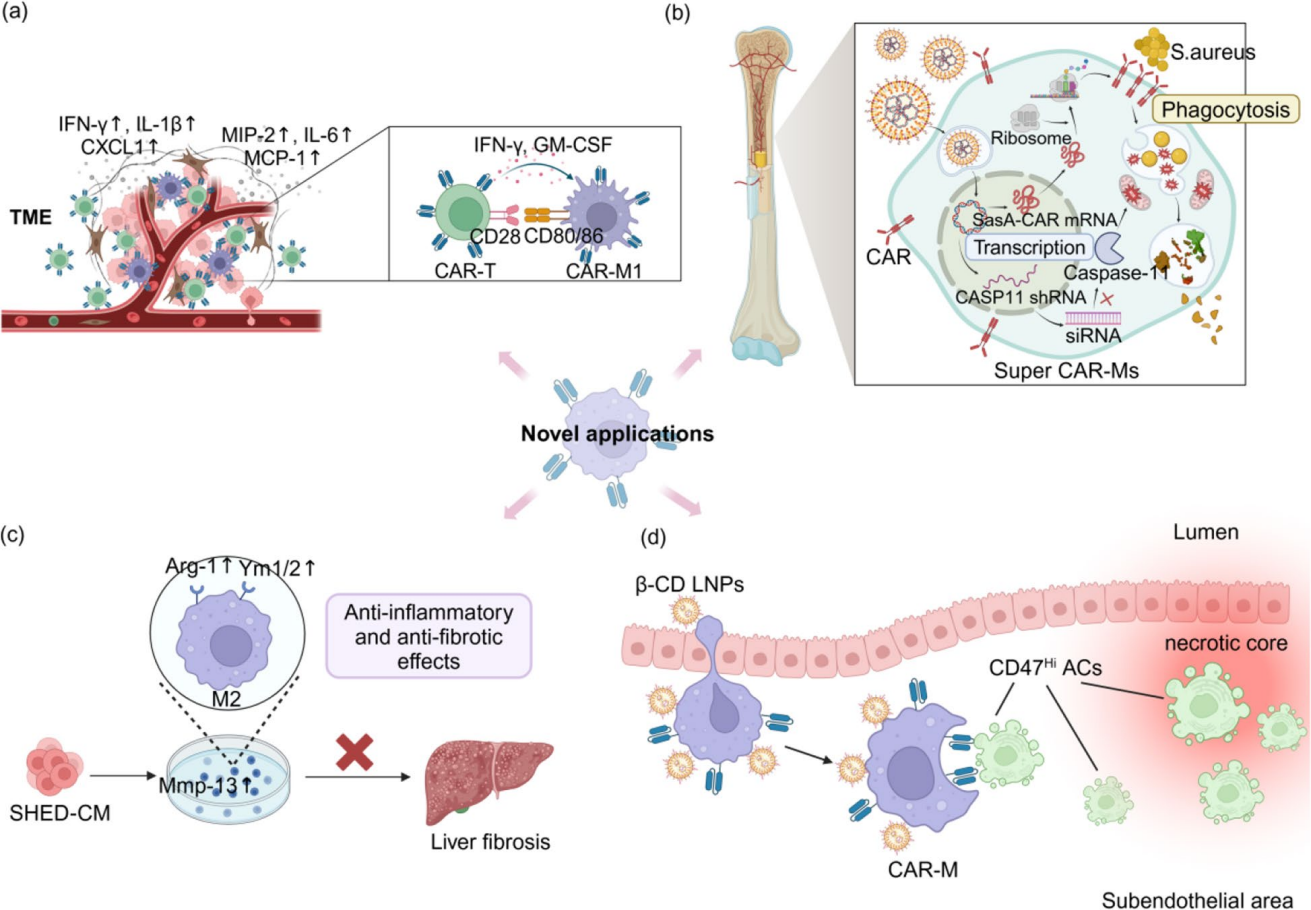
Innovative CAR-M therapies. **a** CAR-M and CAR-T can synergistically induce the death of tumor cells. IFN-γ and GM-CSF secreted by CAR-T cells not only promoted the transformation of macrophages to M1, but also improved the expression of CD80/86, thereby feedback-activating CAR-T activities. Moreover, their collaboration leads to elevated levels of inflammatory cytokines, fostering a conducive environment for anti-tumor immune responses. **b** Nanoparticle coating transferred CAR genes targeting S. aureus into macrophages to generate CAR-M, while simultaneously silencing Caspase-11 to facilitate the recruitment of mitochondria to the phagosome, increasing the killing ability of macrophages. These super CAR-Ms efficiently eradicated S. aureus and triggered robust bactericidal immunologic responses at the bone-implant interface. **c** Macrophages treated with SHED-CM exhibited characteristics of the M2 phenotype, a special phenotype known for its anti-inflammatory and anti-fibrotic effects.This observation suggests that CAR-M could also be utilized for the treatment of inflammatory diseases. **d** To mitigate atherosclerosis, CAR-Ms were engineered to selectively target CD47^Hi^ ACs, thereby enhacing cell clearance and diminishing inflammation. Furthermore, the use of LNPs containing HPβ-CD increased the phagocytic capacity of CAR-M toward ACs

CAR-M and CAR-T can synergistically kill tumor cells in vitro [109] (Fig. 4a). Cytokines derived from CAR-T cells, such as IFN-γ and GM-CSF, have been shown to convert macrophages into a M1 phenotype, augmenting the cytotoxicity of CAR-M [110]. Simultaneously, these inflammatory factors secreted by CAR-T cells can trigger the upregulation of CD80 or CD86 on CAR-M, which may, in turn, positively modulate the cytotoxic capacity of CAR-T cell via CD28 activation [109]. Moreover, the experiment revealed that the levels of cytokines, like IFN-γ, IL-1β, CXCL1, maximal implantation potential (MIP)-2, IL-6 and monocyte chemotactic protein (MCP)-1, drastically increased when CAR-T and CAR-M were cocultured, potentially shaping an inflammatory TME in vivo. The optimistic synergistic effects of CAR-T and CAR-M suggest the feasibility of combination therapy for cancer treatment. In addition, the tumor infiltration ability of CAR-M may recruit more CAR-T cells into the tumor site and exert robust tumor killing ability. However, attention should be paid to the occurrence of a cytokine storm [111].

Besides targeting tumor cells, an implant nanoparticle coating has been reported to transfer CAR genes targeting S. aureus surface protein A (SasA) into macrophages, thereby generating CAR-M to prevent periprosthetic joint infection [74] (Fig. 4b). Intracellular Methicillin-resistant Staphylococcus aureus (MRSA) would trigger Caspase-11 activation to prevent the recruitment of mitochondria to the vicinity of the phagosome, and thus escaping intracellular killing by ROS [112]. Concurrently CASP11 shRNA was introduced into macrophages to pruduce super CAR-Ms, which facilitated the recruitment of mitochondria to surround phagosomes containing phagocytic bacteria [112]. These super CAR-Ms efficiently eradicated S. aureus and triggered robust bactericidal immunologic responses at the bone-implant interface. Recently, Tang et al. developed CRV peptide-modified lipid nanoparticles to assist with the delivery of MRSA-targeted CAR mRNA and CASP11 siRNA [113], providing a therapeutic platform for sepsis.

Compared to proinflammatory phenotyped CAR-M in tumor therapy, a novel non-tumor treatment that depends on tissue remodeling and anti-inflammatory functions of macrophages has been developed (Fig. 4c). After the treatment of conditioned medium from stem cells derived from human exfoliated deciduous teeth (SHED-CM), macrophages exhibited characteristics indicative of the M2 phenotype, including high expression of Arg-1 and Ym-1. Repeated intravenous administration of these macrophages significantly ameliorated liver fibrosis and inflammation in a murine NASH model, inhibiting liver cell apoptosis and reducing the activation of inflammatory macrophages. CAR-M can also be programmed into a M2 phenotype. Studies have suggested that adoptive transfer of anti-inflammatory macrophages and CAR-M could mitigate the progression of inflammatory diseases [114], presenting a brand-new platform to propel cellular engineering. Furthermore, CAR-M targeting urokinase plasminogen activated receptor (uPAR) exhibited potent phagocytosis of hepatic stellate cells (HSCs), suggesting its potential for the treatment of patients with liver fibrosis [75].

The removal of dying cells, or efferocytosis, is indispensable in the resolution of inflammation, especially in atherosclerosis. A CAR-macrophage that can target and engulf phagocytosis-resistant apoptotic cells expressing CD47 is developed to mitigate the risk of secondary necrosis in atherosclerosis [80] (Fig. 4d). Furthermore, to resolve the removal of cholesterol crystals, hydroxypropyl β-cyclodextrin (HPβ-CD) is employed in the LNP to improve cholesterol solubility, which is shown to increase free cholesterol conversion to oxysterols through the liver X receptor (LXR) pathway and promoting cholesterol efflux [115]. LNPs containing HPβ-CD not only improved the in vivo bioavailability of β-cyclodextrin, but also enhanced the macrophage phagocytosis of ACs. The study demonstrated that the synergistic effect of CAR-Ms and LNPs can significantly enhance the efferocytosis of CAR-Ms, presenting a potential therapeutic approach for atherosclerosis [80].

## Conclusions and perspective

Taken together, macrophages distinctly play a pivotal role in anticancer functions, serving not only as phagocytic machinery but also as antigen presenters, TME modifiers, T-cell killing facilitators and immune stimulators. To date, CAR-M therapy has demonstrated promising results in mouse models. Given the outstanding efficacy of CAR-M strategy in animal experiments, a growing number of clinical trials have been approved by FDA, with six currently in progress and one terminated. In 2018, MCY-M11 (NCT03608618) was administrated to patients with ovarian cancer and peritoneal mesothelioma with mesothelin expression. In 2020, TAK-102 (NCT04405778) targeting GPC3 for the treatment of GPC3-expressing solid tumors. In 2021, CAR-M targeting HER-2 entered clinical trials for patients with relapsed/refractory HER-2 positive solid tumors and breast cancer (NCT04660929 CT-0508 & NCT05007379). In 2022, TAK-103 (NCT05164666), which also targeted mesothelin, was able to attack mesothelin-expressing advanced or metastatic solid tumor cells. By 2024, two additional clinical trials are underway: CT-0525 (NCT06254807) and human HER-2 targeted CAR-M (NCT06224738), aimed at treating HER-2 over-expressing solid tumors and HER2-positive advanced gastric cancer with peritoneal metastases, respectively. Although the final results of these clinical trials have yet to be announced, they are anticipated to provide valuable insights for the next steps in CAR-M research, paving the way for more CAR-M therapeutic strategies.

However, the safety of CAR-M therapy still cannot be ignored. IL-6 released by macrophages participates in the occurrence of CRS to a large extent. Some studies have displayed that the structure of CAR may have a certain degree of influence on the level of IL-6; specifically, mice treated with CAR-147 macrophages exhibited lower IL-6 levels compared to those with CAR-iMac involvement, which resulted in much higher IL-6 levels [8]. Further research is needed to figure out the relationship between CAR structure and cytokine levels. After all, tumor microenvironment in humans is more complex due to dynamic interactions between immune cells and tumor cells, making the immune effects of CAR-M on humans uncertain [116]. Nevertheless, there is no denying that CAR-M therapy has the potential to bring more benefits to patients with different diseases, and there is considerable room for further development.

## Data Availability

All data was included in the manuscript

## Abbreviations

ADCC: Antibody dependent Cellular cytotoxicity
ADP: Antibody dependent phagocytosis
Arg1: Arginase 1
AZA: 5-Azacytidine
ACs: Apoptotic cells
β2M: β2-Microglobulin
CAR: Chimeric antigen receptor
CARM: CAR-macrophage
CpG: Cytosine-phosphate-guanine
CRS: Cytokine release syndrome
DFMO: α-Difluoromethylornithine
DNMT: DNA methyltransferase
ECM: Extracellular matrix
GVHD: Graft-versus-host disease
GPC3: Glypican-3
hPSCs: Human pluripotent stem cells
HSCs: Hepatic stellate cells
HCC: Hepatocellular carcinoma
HPβ-CD: Hydroxypropyl β-cyclodextrin
iPSCs: Induced pluripotent stem cells
ITAM: Immunoreceptor tyrosine-based activation motif
LILRB1: Leukocyte immunoglobulin like receptorsubfamily B member 1
LXR: Liver X receptor
LNPs: Lipid nanoparticles
MARCO: Macrophage receptor with collagenous structure
MHC: Major histocompatibility complex
MMPs: Matrix metalloproteinases
MRSA: Methicillin-resistant Staphylococcus aureus
NO: Nitric oxide
NRP1: Neuropilin-1
ODC: Ornithine decarboxylase
ROS: Reactive oxygen species
SHED-CM: Conditioned medium from stem cells derived from human exfoliated deciduous teeth
SasA: S. aureus surface protein A
TAAs: Tumor associated antigens
TAMs: Tumor-associated macrophages
TCR: T cell receptor
TME: Tumor microenvironment
uPAR: Urokinase plasminogen activated receptor
VEGFR2: Vascular endothelial growth factor receptor-2

## Data availability

No datasets were generated or analysed during the current study.
